# The Generation of an Engineered Interleukin-10 Protein With Improved Stability and Biological Function

**DOI:** 10.3389/fimmu.2020.01794

**Published:** 2020-08-11

**Authors:** Faisal Minshawi, Sebastian Lanvermann, Edward McKenzie, Rebecca Jeffery, Kevin Couper, Stamatia Papoutsopoulou, Axel Roers, Werner Muller

**Affiliations:** ^1^Department of Laboratory Medicine, Faculty of Applied Medical Sciences, Umm Al-Qura University, Makkah, Saudi Arabia; ^2^Lydia Becker Institute of Immunology and Inflammation, University of Manchester, Manchester, United Kingdom; ^3^Centre for Translational Medicine, Thomas Jefferson University, Philadelphia, PA, United States; ^4^Manchester Institute of Biotechnology, Faculty of Science and Engineering, University of Manchester, Manchester, United Kingdom; ^5^Department of Cellular and Molecular Physiology, University of Liverpool, Liverpool, United Kingdom; ^6^Institute of Immunology, Medical Faculty Carl Gustav Carus, University of Technology Dresden, Dresden, Germany

**Keywords:** interleukin-10, immunoregulation, inflammation, cytokine, covalent linker, stable dimer

## Abstract

Interleukin-10 (IL-10) is an immunoregulatory cytokine that plays a pivotal role in modulating inflammation. IL-10 has inhibitory effects on proinflammatory cytokine production and function *in vitro* and *in vivo*; as such, IL-10 is viewed as a potential treatment for various inflammatory diseases. However, a significant drawback of using IL-10 in clinical application is the fact that the biologically active form of IL-10 is an unstable homodimer, which has a short half-life and is easily degraded *in vivo*. Consequently, IL-10 therapy using recombinant native IL-10 has had only limited success in the treatment of human disease. To improve the therapeutic potential of IL-10, we have generated a novel form of IL-10, which consists of two IL-10 monomer subunits linked in a head to tail fashion by a flexible linker. We show that the linker length *per se* did not affect the expression and biological activity of the stable IL-10 molecule, which was more active than natural IL-10, both *in vitro* and *in vivo*. We confirmed that the new form of IL-10 had a much-improved temperature- and pH-dependent biological stability compared to natural IL-10. The IL-10 dimer protein binds to the IL-10 receptor similarly to the natural IL-10 protein, as shown by antibody blocking and through the genetic modifications of one monomer in the IL-10 dimer specifically at the IL-10 receptor binding site. Finally, we showed that stable IL-10 is more effective at suppressing LPS-induced-inflammation *in vivo* compared to the natural IL-10. In conclusion, we have developed a new stable dimer version of the IL-10 protein with improved stability and efficacy to suppress inflammation. We propose that this novel stable IL-10 dimer could serve as the basis for the development of targeted anti-inflammatory drugs.

## Introduction

IL-10 is a pleiotropic cytokine that is produced by different cell types, including myeloid cells (dendritic cells, macrophages, eosinophils, neutrophils, and mast cells) and lymphoid cells (NK, B cells, and T cells) with broad anti-inflammatory activity. Macrophages and myeloid dendritic cells express IL-10 upon activation of MyD88 and TRIF-dependent TLR pathways such as TLR3 and TLR4, by stimulation with dsDNA and LPS, respectively (1). Moreover, tolerogenic dendritic cells (CD11c^low^CD45RB^high^) produce large amounts of IL-10 in response to LPS, which induces T regulatory cell development (2, 3). Natural regulatory cells (nTreg) produce IL-10 in the response to IL-2, which is vital for immune homeostasis (4).

Structurally, IL-10 belongs to the class II cytokine family, which involves IL-19, IL-20, IL-22, IL-24 (Mda-7), IL-26, and interferons (IFN-α, -β, and -γ) (5). The IL-10 is a member of helical cytokines as an IL-10 monomer consists of six α-helices (A-F) (6). Biologically active IL-10 is a non-covalent homodimer, which is described as a three-dimensional (3D) domain swapping protein with a molecular mass of 37 kDa (18.5 kDa for each one) (7–9). The IL-10 receptor (IL-10R) is a member of the class II cytokine receptor family and consists of two subunits, IL-10R1 and IL-10R2 (10, 11). IL-10 binds to IL-10R with high affinity; however, it can be species-specific. For example, mouse IL-10 was able to block the binding of human IL-10 to mouse cells but not human cells (12). Analysis of the protein crystal formed of IL-10 bound to soluble IL-10R1 revealed that the 3D domain-swapped homodimer IL-10, which consists of helices E and F from one chain inserted into the hydrophobic cleft formed by into helices A–D of the other chain, is essential for receptor-binding (13). IL-10 binding to the IL-10R complex activates a Janus kinase- Signal Transducers and Activation Transcription system (JAK/STAT) signaling pathway. IL-10/IL-10R promotes phosphorylation and activation of the transcription factor STAT3, which is required for the IL-10 immunoregulatory effect (14).

The anti-inflammatory activity of IL-10 is in part due to the inhibition of the synthesis of pro-inflammatory cytokines such as tumor necrosis factor (TNF) (15). Furthermore, IL-10 downregulates MHC class II expression and helps to promote wound healing (5, 16). The IL-10 showed to have an immunoregulatory effect during an infection with *Toxoplasma gondii* (17), *Mycobacterium* spp. (18), *Herpes simplex virus* (19), and malaria (20) by ameliorating the exaggerated T helper 1 and CD8^+^ T cells response including. A defect of IL-10 or the IL-10 receptor has been linked to excessive immune reactions and a disposition to chronic inflammatory disease, such as the early onset of inflammatory bowel disease (21–23). Also, changes of the gut microflora could lead to a change in the regulation of the gut-associated immune system, resulting in chronic gut inflammation, which in part could be the result of dysregulated IL-10 expression (21, 24).

Here we report the generation of a new form of IL-10 more suitable for therapeutic intervention, as the natural IL-10 has only a short half-life *in vivo*. The stability of the non-covalent IL-10 dimer strongly depends on physical parameters such as temperature and pH. The IL-10 dimer dissociates to a monomeric form at low protein concentrations or at acidic pHs, as typically found in inflamed tissue. Acidic pH has been found, for instance, in fracture-related hematomas (ranging as low as pH 4.7), in cardiac ischemia (pH 5.7) (25). Examinations of inflamed skin showed pH values of 5.8–7.2 (25–27). It has been demonstrated that ~10 and ~50% of human IL-10 was dissociated (i.e., decayed) when heated to 37 and 55°C, respectively, for 1 h (9). Moreover, to date, the free IL-10 monomer has not been found in the solution. The IL-10 monomer could not exist in solution due to the presence of significant hydrophobic residues, which are shielded by interaction involved in the dimer form (6). Therefore, we reasoned that the generation of a stabilized dimer form of IL-10 might be a promising approach to overcome the inherent IL-10 instability and thereby improve its therapeutic potential.

One way to improve the stability of the IL-10 structure has been proposed by generating a stable IL-10 dimer using an internal flexible linker (28). Expressing the dimer as a single continuous fusion protein in which the monomers are connected by a flexible linkers (Gly-Ser) may offer a stability advantage and improve biological activity (29). Moreover, Gly-Ser linkers in recombinant proteins could play a general role in overall stability and solubility. A previous study has shown that using a Gly-rich linker to tether the dimeric forms of HIV-1 proteases (HIV-PR) results in a more stable form compared to the natural HIV-PR at pH 7.0 (30). Moreover, Foss *et al*. showed that using flexible linkers (Gly-rich linker) in transthyretin, the carrier of the thyroid hormone, is more stable than the natural after urea treatment (31). We now generated a functional IL-10 by linking two monomers using a flexible linker. We examined and compared the biological activity of the stable IL-10 dimer against the natural or non-covalently linked IL-10, both *in vitro and in vivo*. Here, we demonstrate that novel recombinant IL-10 dimer has improved stability and higher biological activity; therefore, it has the potential to be a building block for future IL-10 based immunotherapy treatment regimes.

## Methods

### Plasmid Design and IL-10 Construct

The eukaryotic expression vector pCEP V19 was used to express the cDNA of natural mouse IL-10 (Nm) and stable mouse IL-10 (STm). The C-terminus of pCEP V19 includes Factor XA (FXA), human serum albumin (HSA), thrombin. oriP: replication origin of Epstein-Barr virus, EBNA-1: Epstein-Barr virus nuclear antigen-1, ampicillin: ampicillin resistance gene (β-lactamase), pUC ori: the bacterial origin of replication, puromycin: puromycin resistance gene, CMV: Promoter of cytomegalovirus, BM40: a signal sequence of protein BM40, 8 His tag: 8 histidine residues, thrombin: thrombin, NheI, Bsu36I, BamHI: restriction sites, mouse IL-10 cDNA, SV40 pA: polyadenylation signal of SV40 Virus. The expression vector was generated and provided to us by Manuel Koch (Institute of Biochemistry II, University of Cologne). The nine amino acids (-G_3_SG_4_S-) and 13 amino acid linker(-G_3_SG_4_-SG_4_-) were generated by inserting the synthetic insert into the second monomer of mouse IL-10. The stable IL-10 cDNA was digested with the BamHI enzyme before ligating the synthetic inserts containing a nine and 13 amino acid linker with 5′ BamHI restriction site and 3′ BglII restriction sites. The confirmation of the correct insert orientation was carried out using NheI and BamHI restriction digestion. The stable human IL-10 (STh), and mutant cDNAs of mouse IL-10 were synthesized (GeneArt Gene Synthesis: Thermofisher). Oligonucleotides were cloned into pMA-RQ, Col E1 origin: the bacterial origin of replication, ampicillin: ampicillin resistance gene (β-lactamase) (Thermofisher), then subcloned into the eukaryotic expression vector pCEP V19.

### Transfection of HEK_293_EBNA, IL-10 Expression, and Purification

HEK_293_EBNA (HEK) mammalian cell line was generated from human embryonic kidney cells. The cell line was maintained in Dulbecco's modified Eagle's medium (DMEM) with 4.5 g/L glucose, 10% fetal bovine serum (FBS) (Life Technologies Ltd), 1% L-Glutamine, and 1% Penicillin-Streptomycin (Pen-Strep) (Sigma). HEK cells carry the Epstein-Barr Virus Nuclear Antigen-1 (EBNA-1) gene and allow for improved protein production since plasmids replicate competently in positive cells. HEK cells were transfected using Lipofectamine 3000 (Invitrogen). HEK cells were first seeded at 1 × 10^6^ cells/well in a 6-well plate. Upon reaching a confluence of ~80–90%, 5 μg of the plasmid DNA was mixed with different volumes of lipofectamine 3000 (3.5 and 7.5 μl) in 125 μl of Opti-MEM™ (Thermofisher), and the mixture was incubated for 15 min at room temperature. Afterward, the lipo-DNA mixture was added dropwise onto the cells. After 24 h, the cells were washed once with PBS, and selection media containing puromycin (Gibco) was added (DMEM, 10% FBS, 1% Pen-Strep, 1% of L-Glutamine, (2 μg/mL) and incubated for 3 days. The cells were then washed with PBS before adding fresh medium minus puromycin. The supernatant was harvested and stored at −20°C until further use. The transfected cell supernatant was loaded at 4°C onto a HisTrap HP column (GE Healthcare). The column was then washed with 10 CV of binding buffer, followed by stepwise elution of the protein with increasing imidazole concentration (50, 100, 250, and 500 mM) in binding buffer. Protein content and purity of each fraction were visualized by Coomassie staining. Positive fractions were pooled and dialyzed at 4°C against PBS.

### Animal Models

Cells derived from mice that were used in the *in vitro* experiments were housed at the University of Manchester Biological Services Facility (BSF) under specific-pathogen-free conditions. They had easy access to food and water on a 12/12-h light cycle. All breeding mice were routinely screened (3 monthly or annually where applicable) according to BSF recommendations. The mouse strains (hTNF.LucBAC and C57BL/6) were bred in this study under a Home Office project license (70/7800) (P8829D3B4) in agreement with the Animal (Scientific Procedures) Act 1986. The C57BL/6 mice were ordered from Charles River (Charles River Laboratories, Inc., Harlow, UK). The *in vivo* experiments were performed at the University of Cologne, Germany, under animal experimental license 24-9168.11-1/2009-22.

### Purification of Bone Marrow-Derived Macrophages

Mouse bone marrow-derived macrophages cells (BMDMs) were isolated, as described previously (32). Briefly, bone marrow-cells were dissected from femurs and tibiae and plated into a complete media (RPMI 1640 medium (Sigma) supplemented with 10% v/v FBS (Life Technologies Ltd), 100 IU/mL penicillin 100 μg/mL streptomycin (Sigma), 50 ng/mL mouse colony-stimulating factor (MCSF) (Promega), and 50 μM β-mercaptoethanol) (Sigma) at 5 × 10^6^ cells per 90 mm bacterial petri dish (Sterilin, UK) for 4 days. On day 4, 10 mL of complete media was added and incubated for 3 days. Adherent cells were then harvested with 5 mL of PBS supplemented with 5% v/v FBS and 2.5 mM EDTA. For splenocyte isolation, the spleen was homogenized and filtered through nylon mesh filters (70 μM; Becton Dickinson, UK) to generate a single-cell suspension. RBCs were lysed with ammonium chloride potassium (ACK) lysis buffer before the cell pellet was resuspended in DMEM medium supplemented with 10% v/v/FBS, 1% w/v/Penicillin/Streptomycin, 1 mM glutamine and 50 μM β-mercaptoethanol.

### Cell-Based Luciferase Reporter Assay

The cell-based luciferase reporter assay has been previously described (32). BMDMs were plated in 96 well plates (OptiPlate-96, White Opaque 96-well Microplate; Perkin Elmer, UK) at 1 × 10^5^/well in 0.1 mL medium containing 1 mM luciferin (Promega) and left to rest overnight. Cells were stimulated with LPS (*Salmonella enterica* serovar Minnesota R595; Alexis Biochemicals, UK) (10 ng/mL) alone or in the presence of commercial mouse IL-10 mouse (Protech), natural or stable IL-10 proteins. The anti-IL-10R antibody (clone: 1B1.3a) (Biolegend UK Ltd) was used to validate that the alteration in the luciferase response observed was dependent on the IL-10 receptor's engagement with IL-10. Unstimulated cells were used as a negative control. The luciferase activity was measured over time in a CO_2_ Lumistar Omega luminometer (BMG Labtech, UK).

### Temperature- and pH-Dependent Stability Study

Stability experiments were performed as previously described (9), with the biological activity of IL-10 being monitored by luciferase assay. Briefly, 0.1μg/mL of IL-10 sample was incubated at 55°C in time-course (to 30 min). The pre-heated IL-10 sample was added together with LPS on hTNF.LucBAC BMDMs and the luciferase activity was monitored over time. The pH-effect on the biological stability was determined by first pre-incubating a 0.1 μg/mL sample in different pH buffers (sodium citrate pH 2.5, sodium phosphate pH 5.5–6.5 and TRIS-base pH 8.0–10.0) for 24 h at 4°C followed by buffer exchange with PBS using a spin Desalting column (Thermofisher). Protein was diluted to a final concentration in each experiment of 10 ng/mL before testing for biological activity.

### Enzyme-Linked Immunosorbent Assay (ELISA) for IL-10

The recombinant fusion mouse and human IL-10 proteins were detected after purification using Ready Set Go ELISA kits (Cat mIL-10 50-112 eBioscience, UK, Cat hIL-10 88-7106) according to the manufacturer instructions. Briefly, 96-well flat-bottom high-affinity ELISA plates were coated overnight at 4°C with the capture antibody. Plates were washed three times with washing buffer (0.05% Tween 20 PBS) before the addition of blocking buffer to each well with 1X ELISA Diluent (supplied) for 1 h. Standards were prepared and added in 2-fold serial dilutions (4,000–31.25 pg/mL) after washing the plate three times as above. The recombinant protein was diluted 100-fold before serial dilution (1/2) were incubated for 2 h at RT. The detection antibody was added and incubated for 1 h after washing three times with washing buffer as above. Plates were further rewashed three times before incubation with streptavidin-horseradish peroxidase (HRP) for 30 min at RT. Plates were then washed five times with substrate solution, Tetramethylbenzidine (TMB). After incubation for another 15–30 min at RT in the dark, stop solution (2N H_2_SO_4_) was added (25 μl) to each well. Optical density was measured using Versa-Max ELISA Microplate Reader with 450 filter.

### Western Blot

Purified IL-10 was detected by western blot using the anti-mouse IL-10 (JES5-2A5) (eBiosciences). For Splenocytes (5 × 10^6^) were either left unstimulated or were stimulated with either Nm or STm for 24 h After 24 h cells were washed three times with cold PBS and lysed in 0.5 mL of RIPA Buffer (Sigma) containing 5 ul of Protease Inhibitor Cocktail (Sigma) and incubated on ice for 5 min. Samples were then centrifuged at 5,000 × g for 5 min, and the supernatants stored at −20°C. A nanodrop machine was used to determine the concentration of the protein (absorbance at 280 nm) for each sample. The total protein concentration was adjusted in all samples with the addition of a RIPA lysis buffer.

Protein samples were loaded onto 4–12% BIS-Tris Gels (Invitrogen) using running buffer MED SDS (Invitrogen). 24 μl of sample and 6 μl of SDS Sample Buffer (4X) (Thermofisher) were mixed and heated at 80°C for 5 min. 10 μl of electrophoresis marker (Sigma) was used to determine the molecular size. The gel was run at 100 V until the tracking dye reached the bottom of the gel. The gel was removed from the gel cassettes and placed in the nitrocellulose membrane (Bio-Rad) and blotted using the Trans-Blot Turbo Transfer System (Bio-Rad). The blot was then incubated with blocking solution (5% w/v milk in TBS-Tween) for 90 min at room temperature; before incubation with a primary antibody: anti-rat IL-10 (JES5-2A5) (eBiosciences), anti-mouse total STAT3 (Cell signaling), and anti-rabbit p-STAT3(Cell signaling) in 1:1000 in 5% w/v milk in TBS-Tween overnight at 4°C. After that, the blots were washed four times with TBS-Tween for 20 min; before incubated with the secondary antibody (horseradish-peroxidase (HRP)-conjugated goat anti-mouse or anti-rabbit (Cell signaling) at 1:2000 in 5% w/v milk in TBS-Tween for 60 min. The signals were developed using a Western Blot Chemiluminescent Substrate (ECL western blotting substrate (Cell signaling).

### *In vivo* Experiments

Treatment of LPS-induced inflammation of the skin with IL-10: On three consecutive days, 10 μg each of LPS in a volume of 50 μl, with or without varying amounts of Nm or STm, were injected into the flank at the same site. On the 5th day, the tissue surrounding the injection site became removed en bloc and hematoxylin and eosin (H & E) stained. The tissue in 4% formalin was cut to 5 microns thickness using a microtome. After drying the sections onto slides, the deparaffinization and rehydration stages were carried out. The preparations were subsequently stained with Mayer's hemalum solution (Sigma) and eosin (Sigma), dehydrated again, and covered with entellan (Merck). When viewed in the light microsphere, the basic cytoplasm, elastin, and collagen appeared red-orange, the nuclei dark blue, and erythrocytes yellow-orange.

Treatment of Endotoxin Shock by IL-10: 100 μl PBS containing 25 μg LPS (serotype 055: B5) was injected retro-orbitally (i.v) in C57BL/6 mice (8–12 weeks) to indicate the optimum time for TNF synthesis after LPS treatment. To test the efficacy of IL-10 in TNF suppression, C57BL/6 mice were pre-treated with different amounts of either PBS, Nm or STm (2 μg each) for 30 min before injecting LPS (10 μg) At the indicated time points (1.5, 3, or 6 h), blood was taken retro-orbitally, and ELISA used to determine the TNF serum concentration. IL-10^−/−^ mice were given 10 μg LPS together with increasing concentrations of Nm or STm being injected intravenously. After 3 h, blood was withdrawn, and the IL 6 serum concentration determined by ELISA.

### Statistical Analysis

Results were represented as the mean ± standard error of the mean (SEM). Following assessment for normality and equality of variances, statistical inferences on data were performed using one-way, or two-way analysis of variance (ANOVA) followed by unpaired comparisons of treatment means using Dunnett's *post-hoc* test (LPS-treated or vs. LPS+IL-10) used in the luciferase inhibition assay and (untreated or vs. treated) used in the stability study. Differences were considered statistically significant when *p* < 0.05. Luciferase activity represented as area under the curve (AUC) Statistical analyses were performed using GraphPad Prism-7 Software Statistical Package, La Jolla CA; the USA.

## Results

### Generation, Expression, and Purification of a Stable IL-10 Protein Using a Mammalian Expression System

**Figure 1:**
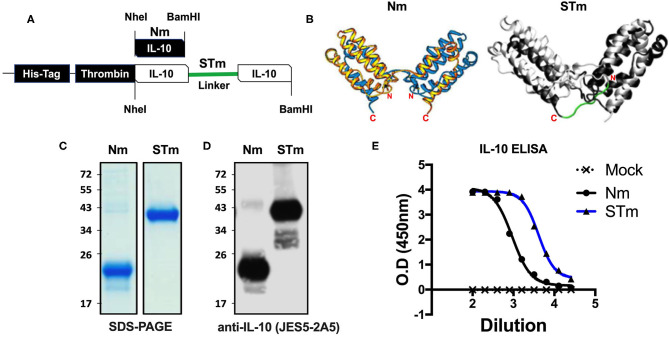
Expression and ELISA measurement of natural and stable mouse IL-10. **(A)** The cDNA construct of both natural mouse IL-10 (Nm) and stable mouse IL-10 (STm). The cartoon illustration of the expected protein folding of both Nm and STm versions. **(B)** The ribbon plot of the natural IL-10 homo-dimer with *N* and *C* terminus obtained from the previous study (13) and the molecular dynamics simulation of stable IL-10. The basis for this simulation is the crystal structure of the hIL-10 dimer (33) (PDB-ID: 2ILK). After about 2 ns of simulated time, the state of the system shown here was found. The peptide linker (-G3SG3-) is colored red. The molecular simulation of stable IL-10 was carried out by Jan-Philip Gehrcke (Biotechnology Center Dresden). **(C)** Both Nm and STm were stained with Coomassie Blue under reducing condition in SDS-page and **(D)** detected by IL-10 antibody in Western Blot. **(E)** Nm (black line) and STm proteins (blue line) were detected ELISA (1/2 dilutions). All data are representative of triplicate wells, and the bars represent standard error of the mean.

The crystal structure of the IL-10 dimer shows that the C-terminus of one monomer is in close proximity to the *N* terminus of the second monomer due to 3D domain swapping with the antiparallel association. This suggested that a stabilized dimer could be generated by linking these two termini. A new recombinant stable IL-10 was generated by cloning two copies of the same IL-10 cDNA in tandem as a continuous polypeptide in the same orientation separated by 7 amino acids linkers (-G_3_SG_3_-) (Figure 1A). Because glycine-serine linkers do not form α-helices and have no reactive side chains (34), they are often used for a flexible and neutral connection of protein domains. Importantly, molecular dynamics simulation (Figure 1B) suggested that the linker would not interfere with the secondary structure of the monomers or IL-10 receptor binding site. In this report, we name the natural IL-10 (non-covalently linked) from mouse as Nm and human as Nh; besides, we name the stable IL-10 dimer from the mouse as STm and human as STh.

Both STm and Nm were generated from HEK cells with a His-tagged pCEP V19 expression vector. Recombinant Nm and STm proteins from parental vector-transfected cells were subjected to purification by an N-terminal His-tag purification column and analysis by SDS-PAGE and Western blotting. Under reducing conditions, the Nm migrated as a monomeric band in the region of 23 kDa, while the STm as a stable dimer migrated as a dimer band of ~41 kDa in SDS-PAGE (Figure 1C). This result corresponds to the calculated molecular weights of 18.7 for IL-10 monomers and 37.3 kDa for IL-10 dimers, respectively (35). Both proteins were also detected using a specific IL-10 antibody (clone: JES5-2A5) in the western blot with different migration profiles due to the monomeric and dimeric forms (Figure 1D). Finally, both STm and Nm were detected by IL-10-ELISA to determine the recombinant IL-10 protein concentrations (Figure 1E) with a commercially sourced IL-10 protein (CmIL-10) (PeproTech) being used as an internal standard.

### The Biological Activity of Stabilized Mouse IL-10 Dimer *in vitro*

**Figure 2:**
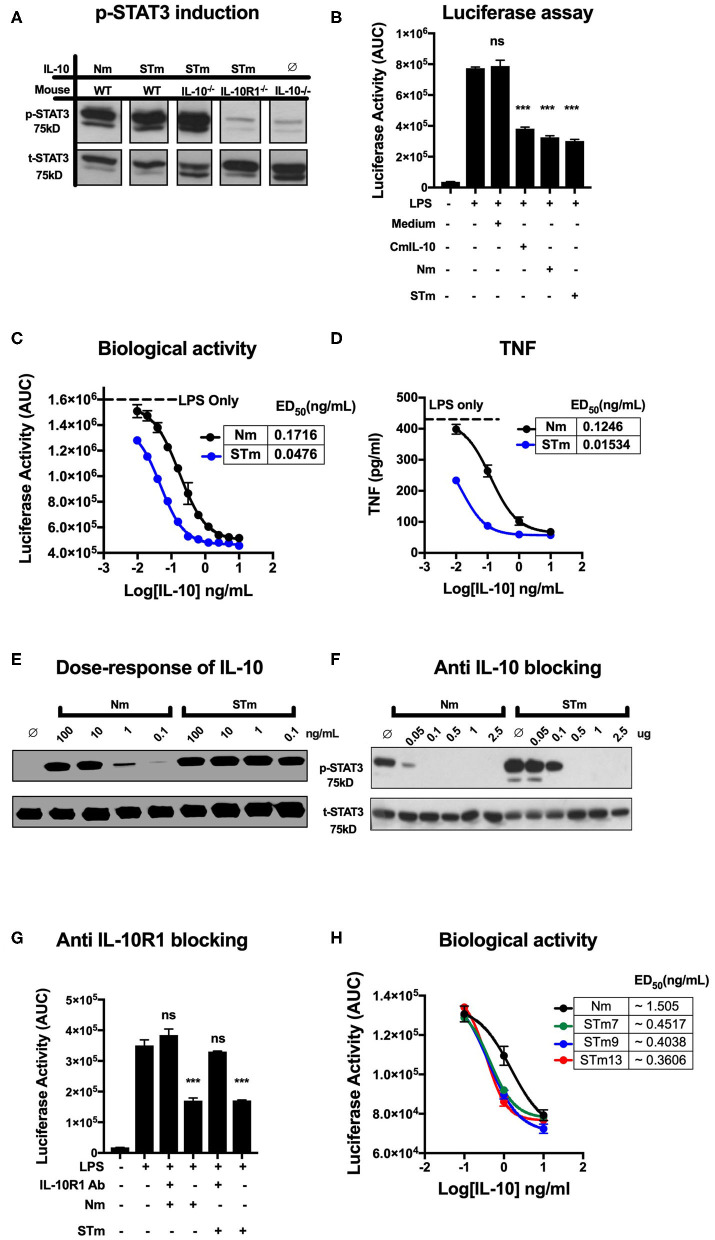
Stable mouse IL-10 (STm) is biologically active *in vitro*. **(A)** Specific induction of STAT3 phosphorylation by STm (5 ng/mL) was verified by the use of spleen cells from different mouse lines: Wild type C57BL/6 (WT), IL-10 knockout (IL-10^−/−^) and IL-10 receptor knockout (IL-10R1^−/−^). **(B)** Luciferase activity was monitored as the area under the curve (AUC) from BMDMs reporter mouse; cells were either unstimulated or stimulated with LPS (10 ng/mL) in the presence or absence of IL-10 from HEK_293_ EBNA supernatants. A commercial mouse IL-10 (CmIL-10) was used as a positive control, and un-transfected cells supernatants (Medium) served as a negative control. Significant difference considered by comparing to LPS stimulated cells as follows: not significant (ns), ***p < 0.001; ANOVA. **(C)** The dose-response effect of IL-10 on LPS-treated BMDMs of the transgenic mouse: the ED_50_ of Nm (black line) and STm (blue line) was calculated as 0.17 and 0.04 ng/mL, respectively. The maximum luciferase induction was determined by treating the BMDMs of the transgenic mouse with LPS alone at 10 ng/mL as it showed a black dot line. **(D)** Soluble mouse TNF was measured from the medium 24 h from BMDMs of the transgenic mouse either after LPS stimulation alone or after co-treating with either Nm (black line) or STm (blue line) in a dose-dependent fashion. **(E)** Splenocyte lysates of the C57BL/6 mouse were either unstimulated (∅) or stimulated with IL-10 (Natural type or Stable) in a dose-dependent manner. **(F)** Inhibition of Nm and STm induced STAT3 phosphorylation by a blocking IL-10 antibody (clone JES5-2A5). IL 10 (2.5 ng) was mixed with the indicated amounts of antibody in one volume of 25 μl of medium and preincubated on ice for 30 min before adding this batch to 475 μl of spleen cell suspension. **(G)** The Luciferase activity represented as AUC from LPS-induce cells from transgenic mouse were either unstimulated or treated with 0.2 μg/mL of the antiIL-10 receptor (antiIL-10Ra) antibody (clone 1B1.3a) for 30 min at 37°C before treatment with LPS (10 ng/mL) or LPS (10 ng/mL), Nm (10 ng/mL), or STm (10 ng/mL). Furthermore, a significant difference is also calculated to compered Nm with STm after antiIL-10Ra treatment; ANOVA. **(H)** The dose-response stable IL-10 on LPS-treated BMDMs of transgenic mouse: the ED_50_ of Nm (black line) is 1.5 ng/mL and the ED_50_ of STm proteins: STm7 (green) STm9 (blue line) and STm13 (red line) is calculated as 0.45 ng/mL and 0.40 and 0.38 ng/mL respectively. All data are representative of three independent experiments, with triplicate cultures per experiment (*N* = 3, *n* = 3), and bars represent standard error of the mean.

Different approaches were used to measure the biological activities of the various versions of the IL-10 proteins we generated. First, we determined IL-10 activity by detection of STAT3 phosphorylation in IL-10 treated lymphocytes. To demonstrate proof of concept, spleen cells from wild-type, IL-10^−/−^, and IL-10R1^−/−^ mice were prepared and stimulated with the purified IL-10 proteins (5 ng/mL). Phosphorylated STAT3 (p-STAT3) (75 kDa) was detected by western blot analysis of cell lysates. Both STm and Nm induced p-STAT3 in murine wild-type and IL-10^−/−^ but not in IL-10R1^−/−^ receptor-deficient cells (Figure 2A), demonstrating that the STm IL-10 uses the IL-10R for signaling.

We compared regulatory activities of STm, Nm, and commercial mouse IL-10 (CmIL-10) using BMDMs isolated from the hTNF.LucBAC reporter mouse reports the activation of the human *TNF gene* promoter, as previously described (32). STm, Nm, and CmIL-10 (all at 10 ng/mL) significantly suppressed the LPS-induced luciferase production by about 60% (*p* < 0.001 ANOVA) (Figure 2B) Subsequently, we titrated STm and Nm IL-10 and determined half-maximal suppression of luciferase induction (ED_50_) values of 0.04 and 0.17 ng/mL for STm and Nm, respectively (Figure 2C). For validation of this result, we isolated the supernatant of LPS- and IL-10-treated BMDMs after 24 h and measured soluble mouse TNF by ELISA. This alternative readout yielded ED_50_ values in the same range as obtained by quantification by the bioluminescence reporter system (Figure 2D). Thus, STm was 4- to 8-fold more active than natural IL-10. Likewise, Western blot analysis of STAT3 phosphorylation induced by titrating amounts of IL-10 showed about 100-fold higher activity of STm compared to natural mouse IL-10 (Figure 2E). As a further test to quantify the biological activity of STm, we determined concentrations of anti-IL-10 mAb required to inhibit STm-induced STAT3 phosphorylation. While the activity of Nm was almost entirely blocked by pre-incubation with 0.05 mg/ml anti-IL-10 antibody, this antibody concentration did not affect STm activity (Figure 2F).

We believe that the suppression of luciferase is dependent on the IL-10R engagement with IL-10. Therefore, we tested the capability of the IL-10R antibody to block the effect of IL-10 on LPS-BMDMs of h.TNF.LucBAC. Our data showed that the presence of anti-IL-10R1 blocking antibodies completely blocked the biological activity of STm. Our data demonstrate that there is a non-significant change in the luciferase induction of a pre-treated BMDMs with 0.2 μg/mL anti-IL-10 receptors antibody (antiIL-10Ra) compared to the BMDMs treated with LPS only (10 ng/mL) (Figure 2G).

In order to address whether the length of the flexible linker, which may affect solubility, stability, and function of the fusion protein, we generated dimeric IL-10 molecules with different linker lengths -G_3_SG_3_- (STm7), -G_3_SG_4_S- (STm9), and -G_3_SG_4_-SG_4_- (STm13) as described above. Linker length did not impact on the bioactivity of the stable IL-10 as determined by suppression of LPS-induced TNF reporter expression (Figure 2H). Collectively, stable mouse IL-10 shows significantly more potent bioactivity compared to natural mouse IL-10.

### Temperature- and pH-Dependent Stability of Stable Mouse IL-10 Protein

**Figure 3:**
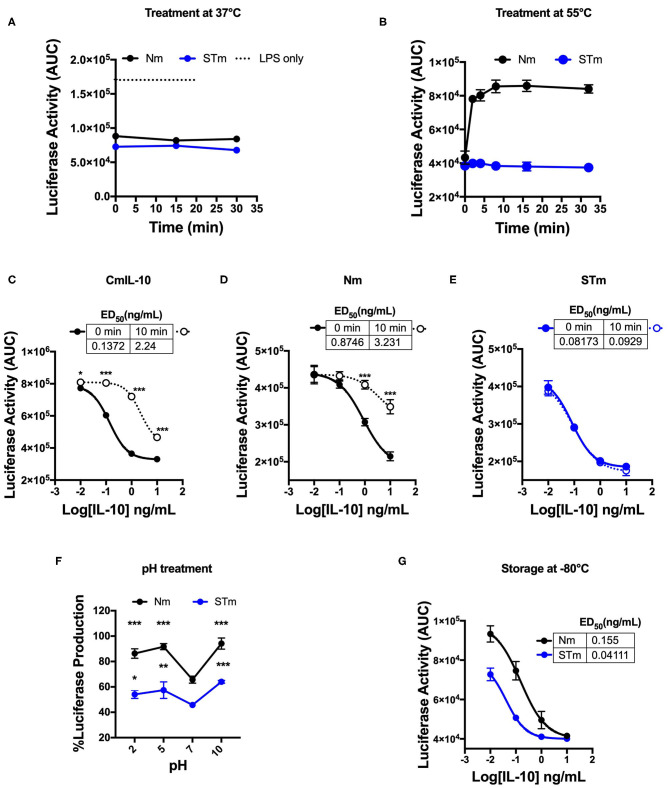
Biological stability of mouse IL-10 variants upon treatment at different temperatures and pH *in vitro*. **(A)** Area Under the Curve (AUC) of luciferase induction was measured after incubating 100 ng/mL Nm (black line) and STm (blue line) at 37°C in the time course. **(B)** Both Nm (black line) and STm (blue line) were treated at 55°C in time course before luciferase activity was measured as the area under the curve (AUC) after LPS-stimulated BMDMs of reporter mouse. **(C–E)** LPS-induced luciferase inhibition was measured after co-stimulated with either heat-treated at 55°C for 10 min (10 min) or untreated (0 min) of the commercial mouse IL-10 (CmIL-10), Nm and STm. CmIL-10 was used as a control in this experiment. The ED_50_ was calculated as follow: **(C)** CmIL-10 heat-treated for (2.24 ng/mL) or untreated (0.137); **(D)** Nm heat-treated for (3.23 ng/mL) or untreated (0.8 ng/mL); **(E)** STm heat-treated for (0.09 ng/mL) or untreated (0.08 ng/mL). **(F)** Both Nm and STm at 100 ng/mL were pre-incubated with different pH buffers at 4°C for 24 h flowed by buffer exchange columns. BMDMs of the transgenic mouse then stimulated with LPS (10 ng/mL), and pH treated IL-10 (Nm and STm) at 10 ng/mL. The percentage of luciferase activities is relative to LPS treatment. The significant difference compared between pH treatments with neutral pH (pH7) of Nm and STm on LPS-stimulated cells ***p < 0.001, **p < 0.005, *p < 0.05; ANOVA. **(G)** To test the effect of the storage of IL-10 protein in −80°C for 6 months, the ED_50_ was calculated and compared between Nm (black line) and STm (blue line). All data are representative of three independent experiments, with triplicate cultures per experiment (*N* = 3, *n* = 3), and bars represent standard error of the mean.

The human IL-10 homodimer was shown to rapidly dissociate into inactive monomers at lower pH and higher temperature (9). We compared the effect of temperature and pH on the biological activity of STm and Nm. A 30 min incubation at 37°C did not affect the capacity of Nm or STm to suppress LPS-induced luciferase expression (Figure 3A). Nm lost bioactivity already upon 5 min exposure to 55°C, whereas the treatment did not affect STm (Figure 3B, Figure S1A). ED_50_ of commercial IL-10 and Nm were significantly reduced by exposure to 55°C for 10 min while STm was not affected by this treatment (Figures 3C–E). Moreover, we investigate the impact of acidic and basic pH on the biological activity of IL-10 protein. Biological activity of both Nm and STm decreased in acidic and alkaline pH compared to neutral pH (pH 7). However, at an acidic pH of 5, STm was significantly more active over a wide concentration range (Figure 3F). We also addressed the effects of freezing and frozen storage on IL-10 bioactivity. Our data indicate that the ED50 of STm IL-10 was 0.041 ng/mL, and ED_50_ Nm was 0.15 ng/mL after storage at −80°C for 6 months (Figure 3G). In summary, we show that stable mouse IL-10 is more resistant to heat and low pH than natural mouse IL-10.

### The Effect of the Site-Specific Mutation on Stable Mouse IL-10 Biology

**Figure 4:**
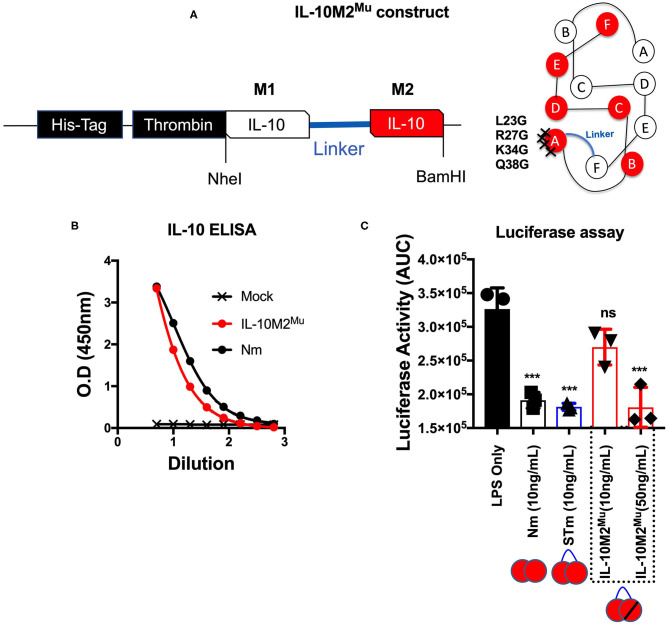
Mutations introduced into one monomer of stable mouse IL-10 at the IL-10R binding site (IL-10M2^Mu^). **(A)** The cDNA construct of both un-mutant IL-10 and IL-10M2^Mu^ was cloned into the expression vector (pCEP V19) and the cartoon illustration of IL-10M2^Mu^ with the location of the 4-points mutations at the IL-10 receptor-binding site on the helix A of IL-10 protein. **(B)** Purified IL-10M2^Mu^ detected by ELISA (1/2 dilutions) to evaluate the expression level. **(C)** Assessment of luciferase suppression after LPS (10 ng/mL) treatment and either co-treated with Nm (10 ng/mL), STm (10 ng/mL), or IL-10M2^Mu^ at (10 and 50 ng/mL). The significant difference is calculated concerning LPS stimulated cells only and symbolized as ***p < 0.001, ns (non-significant). All data are representative of three independent experiments, with triplicate cultures per experiment (N = 3, n = 3), and bars represent standard error of the mean.

We predict that the STm bound to IL-10R like Nm (i.e., natural IL-10). To investigate this, we mutated the IL-10R binding site in the STm dimer. The location of the IL-10R binding site was obtained from the previous study on IL-10/IL-10R interaction (13). For this purpose, we introduced four-point mutations (L23G, R27G, K34G, and Q38G) at the helix A of the second monomer of the STm dimer (Figure 4A). This mutated form of IL-10 named IL-10M2^Mu^. The IL-10 ELISA detected the IL-10M2^Mu^ as validation of the presence of the recombinant protein in culture supernatants of HEK cells (Figure 4B). The luciferase assay, represented as AUC, showed that IL-10M2^Mu^ (10 ng/mL) inhibited the luciferase activity by ~15% (*p* < 0.5, ANOVA). However, 50 ng/mL of IL-10M2^Mu^ inhibited luciferase activity by ~40% as the Nm and STm (*p* = 0.001, ANOVA) (Figure 4C). This data shows that maximal suppression could be achieved with 5-fold of IL-10M2^Mu^ compared to STm and Nm. This experiment may represent the significance of dimerization in STm to retain the maximum activity by binding to the IL-10R as the natural IL-10.

**Figure 5:**
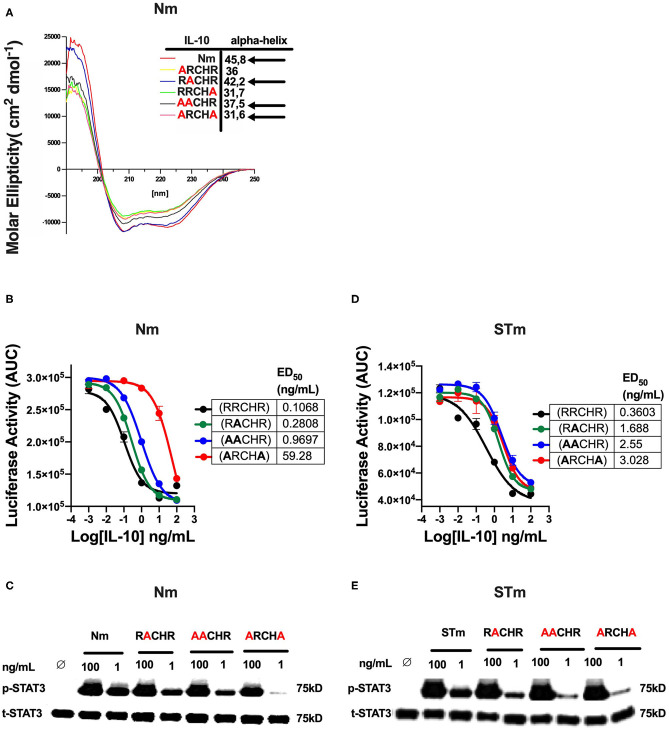
The effect of amino acid substitution at the RRCHR region of mouse IL-10 on biological activities. **(A)** The secondary structure of IL-10 mutants by CD spectroscopy adopted from unpublished data (36). **(B)** The ED50 of natural IL-10 was calculated as flow: Nm^RRCHR^ (un-mutant) (black line) (0.1 ng/mL), Nm^RACHR^ (green line) (0.28 ng/mL), Nm^AACHR^ (blue line) (0.96 ng/mL), and Nm^ARCHA^ (red line) (59.28 ng/mL). **(C)** The activation of cellular signaling (p-STAT3) determines by western blot after lysate from spleen cells of the C57BL/6 mouse treated Nm mutants and un-mutant (control) in dose-response and using the total STAT3 as an internal control. **(D)** The ED50 of stable IL-10 was calculated as flow: STm^RRCHR^ (un-mutant) (black line) (0.36 ng/mL), STm^RACHR^ (green line) (1.6 ng/mL), STm^AACHR^ (blue line) (2.55 ng/mL), and ST^ARCHA^ (red line) (3.02 ng/mL). **(E)** The activation p-STAT3 determines by western blot from spleen cells lysate of the C57BL/6 mouse treated STm mutants and un-mutant (control) in dose-response and using the total STAT3 as an internal control. The data represented as AUC (Area Under the Curve). All data are representative of two independent experiments, with triplicate cultures per experiment (*N* = 2, *n* = 3), and bars represent standard error of the mean.

In previous investigations, we demonstrated that several alanine substitutions in the IL-10^RRCHR^ (i.e., natural IL-10) region have an impact on the structural integrity (α-helical structure) of IL-10 (Figure 5A), with substitution of RRCHR to ARCHA causing the most significant loss of structure (36). Importantly, we found a correlation between changing the degree of α-helical structure with the reduction in the biological activity of Nm, as presented in ED_50_ and confirmed by the p-STAT3 assay (Figures 5B,C). Based on this data, to further investigate the biological properties and the IL-10R binding of the engineered STm, we assessed the corresponding impact of these substitutions on the biological activity of STm. Our data showed that these mutations affected the biological activity of STm to a different extent compared to Nm, as represented in ED_50_ and confirmed by p-STAT3 assay (Figures 5D,E). Specifically, whereas the ARCHA mutation significantly affected Nm biological activity by >500 fold, this only led to a 10-fold loss of function in STm. Conversely, the AACHR and RACHR substitutions that did not dramatically modify STm activity led to a similar loss of function in the STm as the ARCHA mutation. Collectively, the inactivation of one monomer of STm led to a weaker binding to IL-10R; moreover, the impact on the biological activity of amino acid substitution at the RRCHR region was higher in Nm than STm.

### Stabilized Mouse IL-10 Dimer Is Biologically Active *in vivo*

**Figure 6:**
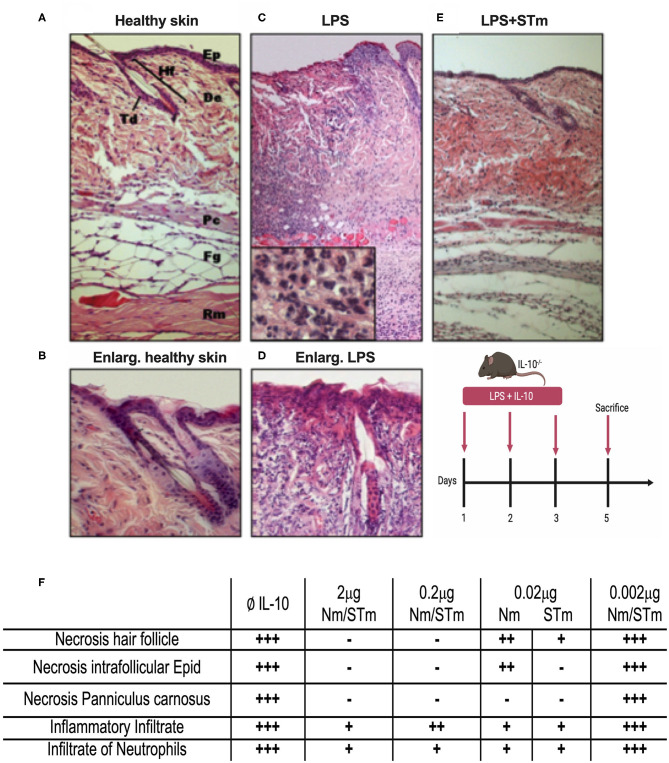
Suppression of LPS-induced dermal inflammation by Nm and STm. **(A)** Overview of healthy skin. Mouse skin consists of a 1–2-layer epidermis (Ep), which forms the hair follicles (Hf) and sebaceous glands (Td) by invagination in the dermis (De). The dermis consists of collagenous connective tissue. This is followed by the muscle layer of the Panniculus carnosus (Pc), the fatty tissue (Fg), and the trunk muscles (Rm). **(B)** Enlargement of healthy skin. The epithelium shows bluish cytoplasm and loose chromatin, as well as two intact hair follicles. **(C)** Overview of necrotic skin. In magnification, a large number of neutrophils is recognizable. **(D)** Enlargement of necrotic skin. The epithelial cells show reddish cytoplasm and condensed chromatin. The hair follicle is dead. **(E)** Overview skin section treated STm (2 μg) STm, which LPS was co-injected. **(F)** A table summarizing the effect of IL-10 (Nm/STm) at different concentrations on LPS-treated (10 μg) skin.

While the above data demonstrated the improved biological activity and stability of STm *in vitro*, it was essential also to examine the efficacy of STm *in vivo*. The efficacy of STm compared to Nm under physiological conditions was first examined utilizing a local inflammatory reaction in the skin of mice. The skin consists of a multilayer structure of the epidermis, as well as the bluish coloration of the basophilic epithelial cytoplasm (Figures 6A,B). Subcutaneous injection of LPS causes a dose-dependent inflammatory infiltration of all layers of the skin (37). Our previous data showed that this reaction is much more severe in IL-10^−/−^ mice, with considerable necrosis of epidermis, dermis, and panniculus carnosus (37). This result indicates the importance of IL-10 in modulating the inflammatory response to LPS in the skin, validating the model to assess the *in vivo* effectiveness of STm in controlling inflammation. Different concentrations of the IL-10 proteins were injected subcutaneously (under the panniculus carnosus) together with LPS (10 μg) into IL-10^−/−^ mice. On 3 consecutive days, an injection was made in the same place in the flank. On the 5th day, the mice were sacrificed, and the tissue removed from the injection site and examined histologically. This experiment showed that injections of LPS without IL-10 resulted in a massive inflammatory local reaction with massive recruitment of numerous macrophages and neutrophils (Figures 6C,D). Moreover, all layers of the skin in the center of the lesion became necrotic compared to healthy skin. The enlargement view (Figure 6D) showed a necrotic hair follicle and necrotic interfollicular epidermis with condensed nuclei and reddish acidophilic cytoplasm as opposed to the basophilic cytoplasm of healthy keratinocytes. Coadministration of STm along with LPS (Figure 6E) suppressed the inflammatory response in a dose-dependent fashion. In Figure 6F, we summarize the biological activities of STm and Nm in protecting LPS induced inflammation. Both STm and Nm were protective at 2 and 0.2 μg. STm appeared to be more effective than Nm at 0.02 μg, where the biological activity of Nm was waning. At 0.002 μg, both Nm and STm had no protective effect against skin inflammation (Figure 6F). This experiment demonstrated the biological effect of STm *in vivo* but did not allow a quantitative comparison between Nm and STm.

**Figure 7:**
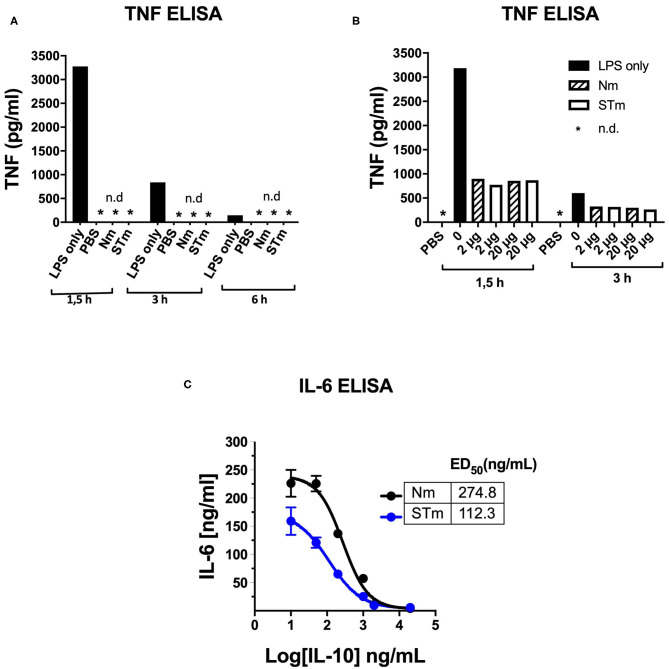
Detection of the *in vivo* activity of the STm by suppression of LPS-induced cytokine release. **(A,B)** 100 μl PBS containing 25 μg LPS i.v. Injected in C57BL/6 mice (8–12 weeks). At the indicated time points, blood was taken retro-orbitally, and ELISA determined the TNF-a serum concentration, *n.d.: not detectable. **(A)** To establish, LPS, PBS, Nm, or STm (2 μg protein each) were injected one at a time. **(B)** 30 min before LPS administration, the indicated amounts of Nm or STm were i.v. Injected. **(C)** IL-10 -/- mice were given 10 μg LPS together with increasing concentrations of Nm or STm i.v. Injected. After 3 h, blood was drawn, and the IL 6 serum concentration determined by ELISA.

The activity of STm was also tested using an *in vivo* model of LPS-induced systemic inflammation. First, the time course of TNF concentration in serum after retro-orbital (i.v.) LPS injection was determined in establishment experiments. A high serum concentration of TNF ~3.2 ng/mL was measurable 1.5 h after the administration of LPS (25 μg i.v.). This value was reduced by about 75% after 3 h and had returned close to baseline levels 6 h after LPS injection. Injections of PBS or Nm and STm (2 μg each) alone did not result in the measurable release of TNF, which could exclude contamination of these reagents with pyrogens (Figure 7A). In the next experiment, different amounts of STm i.v. were injected 30 min before LPS (25 μg i.v.). Here, the STm proved to be highly effective, in a dose-dependent manner, in the suppression of TNF release. The administration of 2 μg Nm and STm reduced serum TNF concentration by ~70% relative to the control mice receiving only LPS. Both at either 2 or 20 μg, Nm and STm were equally effective at suppressing TNF production (Figure 7B). Apparently, in this assay, the maximum level of TNF suppression was reached at 2 μg IL-10, as the injection of 20 mg IL-10 failed to suppress TNF production further. Experiments with titrations of Nm- and STm, however, showed large fluctuations in TNF serum concentration and therefore did not allow a quantitative comparison of the effects of Nm- and STm (data not shown). Therefore, we investigated the ability of Nm and STm IL-10 to inhibit the production of IL-6, which is another cytokine integral within the acute phase inflammatory immune response. The serum concentration of IL-6 was determined 3 h after injection by ELISA. It was found that over a wide dose range, the STm reduced IL-6 release more effectively than Nm. By non-linear regression, Nm suppressed the IL-6 response with an ED_50_ value of 274.8 ng/mL, whereas STm suppressed IL-6 production with an ED50 of 112 ng/mL. Thus, STm was ~2.5-fold (Figure 7C) more potent than Nm. Altogether, the stable form of IL-10 is biologically active *in vivo*, which controls both the local and systemic inflammatory responses.

### The Biological Activity and Stability of Stabilized Human IL-10 Dimer *in vitro*

**Figure 8:**
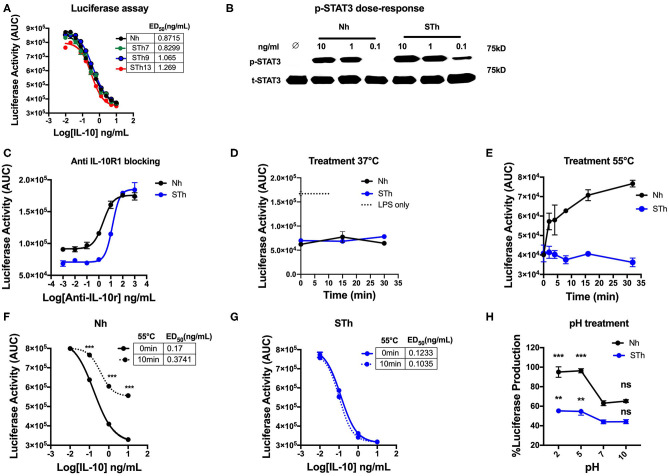
Generation and characterization of the stable version of human IL-10. **(A)** The effect of STh (with different linkers length) and Nh on LPS-induce luciferase activity on BMDMs of transgenic mouse: the ED_50_ of Nh(black line) is 0.87 ng/mL and the ED_50_ of STm proteins: STh7 (green) STh9 (blue), and STh13 (red) is calculated as 0.82, 1, and 1.2 ng/mL, respectively. **(B)** Splenocyte lysates of the C57BL/6 mouse either unstimulated (∅) or stimulated with IL-10 (Natural type or Stable) in a dose-dependent manner. **(C)** The effect mouse antiIL-10 receptor antibody (clone 1B1.3a) on blocking the suppression induced by Nh and STh (10 ng/mL) in a dose-dependent fashion. Furthermore, a significant difference is also calculated to compare Nm with STm after antiIL-10Ra treatment; ANOVA. **(D)** Luciferase activity represented as AUC from LPS-induced BMDMs co-treated with either Nh (black line) and STh (blue line) at 10 ng/mL after incubated at 37°C in the time course. **(E)** Both Nh and STh were incubated at 55°C in time course, and the luciferase activity was calculated after LPS-stimulated and co-treated with either Nm (black line) and STm (blue line) at 10 ng/mL on BMDMs. **(F,G)** LPS-induced Luciferase activity when co-stimulated with either heat-treated at 55°C for 10 min (10 min) or untreated (0 min) of Nh and STh. The ED_50_ is calculated as follow: **(F)** Nh heat-treated for (3.74 ng/mL) or untreated (0.17 ng/mL); **(G)** STh heat-treated for (0.10 ng/mL) or untreated (0.12 ng/mL). **(H)** Both Nh and STh at 100 ng/mL were pre-incubated with different pH buffers at 4°C for 24 h flowed by buffer exchange columns. BMDMs of the transgenic mouse then stimulated with LPS (10 ng/mL) and pH-treated IL-10 (Nh and STh) at 10 ng/mL. The percentage of luciferase activities is relative to the maximum LPS induction of luciferase. The significant difference compared between pH treatments with neutral pH (pH7) of Nh and STh on LPS-stimulated cells; ***p < 0.001, **p < 0.005; ANOVA. All the data above are representative of three independent experiments, with triplicate cultures per experiment (*N* = 3, *n* = 3), and bars represent standard error of the mean.

Our results demonstrated that STm molecules could be generated with potent regulatory activity. Consequently, given the aim of developing an IL-10 based treatment for human disease, we investigated whether it was also possible to create a biologically active and stable version of human IL-10. We first generated and produced a stable human IL-10 using different lengths of the flexible linker (Figure S2A). Our data showed that both Nh and STh, with different linker lengths, are biologically active *in vitro* by suppressing LPS-induced luciferase expression in BMDMs from the h.TNF.LucBAC reporter mice (Figure 8A). STh had a 2.5-fold higher biological activity than Nh, as measured by p-STAT3 activation (Figure 8B), which was confirmed by conventional TNF ELISA assay (Figure S2B). Like the mouse IL-10, the effect of human IL-10 on luciferase suppression was blocked by using mouse anti-IL-10R1 in a dose-dependent manner. Interestingly, our data demonstrate that increased amounts of anti-IL-10R1 antibody were required to block the effect of STh in suppressing luciferase production than to block the effect of Nh (Figure 8C). This data may indicate the binding of STh to mouse IL-10R is of higher affinity compared to the Nh.

In terms of stability, both STh and Nh were incubated during a time course at 37°C, which showed no significant difference in luciferase inhibition (*p* > 0.5, ANOVA) (Figure 8D), which indicates the both Nh and STh are biologically active at physiological temperature. However, Nh gradually lost biological activity after 5 min of heat treatment at 55°C, whereas STh maintained biological activity as measured by luciferase assay (Figure 8E) and TNF-ELISA (Figure S1B). In a dose-response experiment, we also observed that Nh became less potent after heat treatment (55°C for 10 min) (Figure 8F); however, STh maintained suppressive activity (Figure 8G). The pH-dependent stability study showed that STh behave similarly to Nh, but, the STh was more resistant in pH5 and pH2 (Figure 8H). Overall, these data show that the STh is a biologically active protein *in vitro* with higher stability compared to Nh.

**Figure 9:**
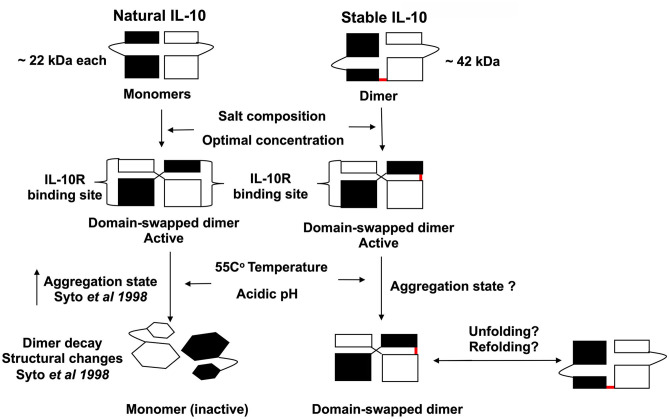
Scheme representing the proposed biological activity and stability of stable IL-10 dimer. Both natural and stable IL-10 are biologically active *in vitro* and *in vivo*. We predict that our stable IL-10 is folded like the natural IL-10 in a domain-swapping fashion. Our mutant IL-10 model demonstrates the importance of dimerization to elicit the maximum inhibitory response. We showed that stable IL-10 is more active after treatment with different physiological stress conditions such as high-temperature and pH compared to natural IL-10.

Overall, we generate a stable IL-10 dimer by linking two IL-10 monomers in a head to tail fashion by a flexible linker (Figure 9). We demonstrate that our novel stable IL-10 dimer is more stable in different stress conditions compared to non-covalently linked IL-10 (i.e., natural IL-10). We assume the stable IL-10 dimer folds as natural IL-10. The IL-10 3D domain-swapped dimerization of IL-10 is essential to form the receptor binding site of the cytokine (13). Our mutational experiment has indirectly proved that stable IL-10 dimer binds and acts by the IL-10 receptor.

## Discussion

Soon after the cytokine IL-10 was cloned, we generated a mouse mutant deficient for IL-10 and could show that these mice developed inflammatory bowel disease that depended on the microflora (21). Based on these findings and subsequent preclinical trials, it was shown that while recombinant IL-10 was safe and well-tolerated in healthy individuals (38), it had limited efficacy in treating inflammatory diseases (39). In this report, we reach an important milestone for the generation of a recombinant IL-10 with more stable biochemical properties. Syto et al. studied the structural and biological stability of non-covalently linked IL-10 dimer under different conditions. It was observed that incubation of human IL-10 dimer for 1 h at 37 and 55°C resulted in the formation of 2 and 22% monomers, respectively (9). By the covalent fusion of two IL-10 monomers, we generated a more stable and most likely also more biologically active form of IL-10 both for mouse and human (Figures 2, 8). The previous study demonstrates that the covalent fusion of dimeric HIV-1 proteases has been shown to improve protein stability compared to natural dimer (30). However, the mechanism of this stability enhancement is not clear. Our possible explanation is that linking the C-terminus of one IL-10 monomer to the N-terminus of the second monomer may increase the stability of the IL-10 3D domain-swapped dimer. The IL-10 3D domain-swapped dimer defined as the exchange of the helices E and F (from the first monomer) into the hydrophobic cleft (helices A-D) of the other monomer, which may not be profoundly affected by the different conditions of stress in stable IL-10 compared to the natural IL-10. This study did not define the potential of refolding stable IL-10 to the known active state after different stress-inducing conditions were applied. We could demonstrate that the new IL-10 dimer binds and acts via the IL-10 receptor, shown that it does not act on IL10R1 deficient lymphocytes (Figure 2A), by blocking the biological activity using anti-IL-10 receptor antibodies (Figure 2F) and by introducing point mutations in the covalent linked IL-10 dimer suggested to be involved in IL-10 and IL-10 receptor interaction (Figure 4). A previous study suggested that IL-10 dimer formation is vital to generate the receptor-binding domain that activates IL-10R signaling via side to side interaction (13). Therefore, it is believed that the correct dimer formation (i.e., 3D domain swapping) in IL-10 is essential to create a receptor-binding domain (13). This hypothesis has been confirmed by generating a stable monomeric form as a model to bind to IL-10R in 1:1 interaction (40). The next step along this line is to generate crystal structures for the new IL-10 proteins and to compare these to the known IL-10 structure (33). Our recombinant stable dimeric IL-10 can now be used as a building block for the development of IL-10 based therapeutic. One of the new features of our stable IL-10 is that we can now prepare asymmetric IL-10 variants as we have control over the complete IL-10 dimer. We can modify each IL-10 molecule in the dimer independently and get 100% variants with a defined order of each of the monomers. This allows in the future more insights into the functional analysis of IL-10 with its receptor and fine-tuning of the biological activity of the stable IL-10 protein.

Using the conditional gene targeting approach, we generated mouse mutants with selective gene inactivation of the IL-10 (41) and the IL-10R (23). Using these mutants in many different settings, we have clear evidence that IL-10 is a local acting cytokine, and there is an apparent cell-type specificity at both at the level of the IL-10 producing and at the level of the IL-10 responding cell. For example, in the T-cell specific IL-10 deficient mouse mutant (41), only T cell-specific responses are dysregulated, while innate responses are unaffected. In contrast, in a macrophage-specific IL-10 receptor-deficient mouse mutant, the T cell responses are unaffected while innate responses are disturbed similarly to IL-10^−/−^ mice. These experiments suggest that the next step toward an IL-10 based therapeutic recombinant protein will be to make an IL-10 protein that can mimic the local acting property of IL-10.This might be achieved by joining our recombinant stable IL-10 to other protein domains that can bind to local regions and/or to specific cell types. Of note, we have generated a fusion protein of IL-10 with an antibody and could see that both the IL-10 activity and the antibody binding property is present in the fusion protein (work in progress). One of the challenging steps in the engineering is to use a form of IL-10 that is less active compared to the natural IL-10 protein in order to allow IL-10 action only when a high local concentration of IL-10 through the local accumulation of the IL-10 protein through the antibody part. Examples of such IL-10 weakening mutations are presented in this report (Figure 4). We presume that we are maybe even able to generate a local acting IL-10 inhibitor, in which one of the two dimers is mutated in a way that the stable IL-10 dimer binds to one of the IL-10 receptor chains and blocking dimerization of the receptor.

In conclusion, the effect of IL-10 in clinical trials is limited due to pleiotropic properties on different cells, and the rapid dissociation of the homodimer at the site of inflammation. Our stable IL-10 protein could be a potential building block for generating a potent and more effective and selective IL-10-based immunotherapy for treating inflammatory diseases and cancers. For instance, a stable IL-10 dimer has already been proposed as a model for generating a target IL-10 immunotherapy because it has a higher biological activity compared to the natural IL-10 monomer (42) and the IL-10 dimer proposed here would allow constructing such cell-type-specific local acting IL-10.

## Data Availability Statement

All datasets generated for this study are included in the article/Supplementary Material.

## Ethics Statement

The animal study was reviewed and approved by Home Office project license (70/7800) (P8829D3B4) in agreement with the Animal (Scientific Procedures) Act 1986 and the *in vivo* experiments were performed at the University of Cologne, Germany, under animal experimental license 24-9168.11-1/2009-22.

## Author Contributions

FM, SL, AR, and WM: conception, design, and stable IL-10 construction. FM and SL: *in vitro* experiment. SL and AR: *in vivo* experiments. FM, SL, and WM: data acquisition. FM, SL, RJ, and EM: IL-10 expression. FM and SP: luciferase assay. FM, SL, SP, KC, AR, and WM: data analysis and interpretation. FM, KC, and WM: drafting of the article. All co-authors: final approval of the manuscript.

## Conflict of Interest

The authors declare that the research was conducted in the absence of any commercial or financial relationships that could be construed as a potential conflict of interest.
