# Supplementary material for: The Generation of an Engineered Interleukin-10 Protein With Improved Stability and Biological Function
Source: Front Immunol. 2020 Aug 11;11:1794. doi: 10.3389/fimmu.2020.01794 (PMC7431522; doi:10.3389/fimmu.2020.01794)
Supplement: Supplementary file 1 [file Data_Sheet_1.docx]

**Supplementary Results**

**
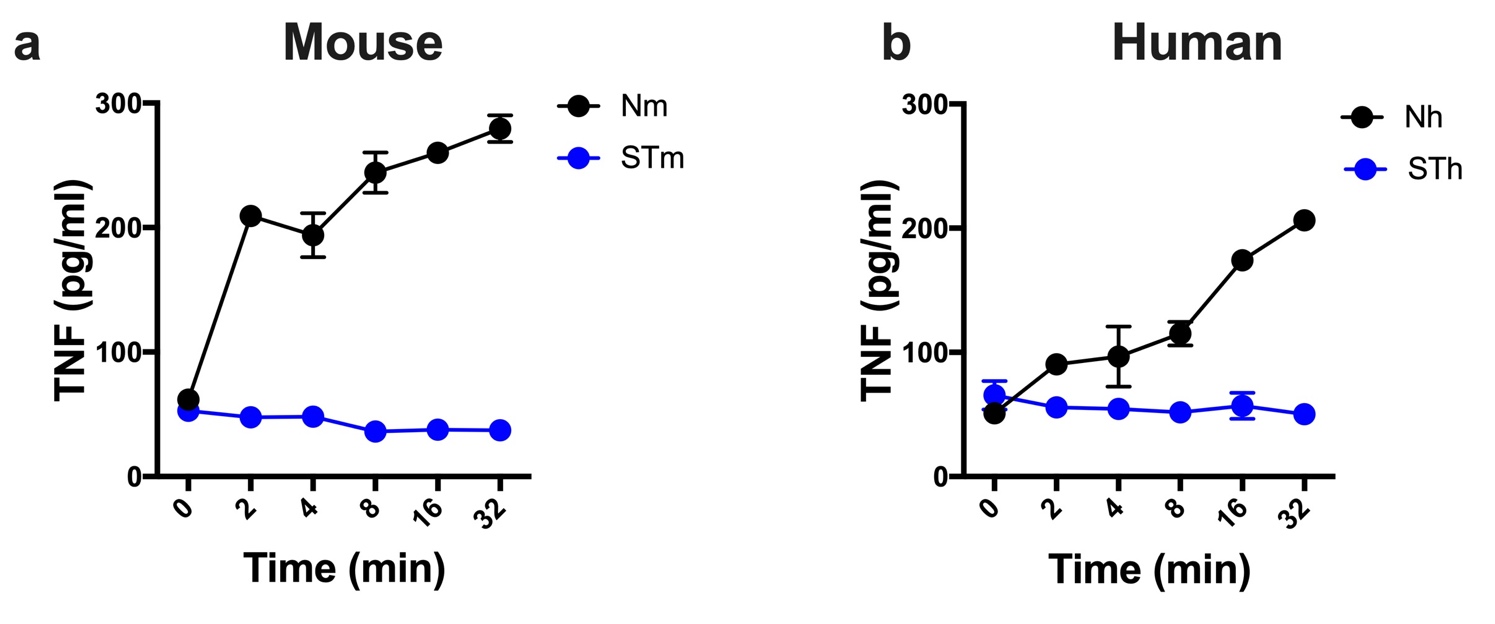
**

**Figure S1. Biological Stability of mouse and human IL-10 upon treatment at different temperatures and pH *in vitro*.** (**a**) Both Nm (black line) and STm (blue line) were treated at 55°C in time course before soluble mouse TNF was measured by ELISA) after LPS-stimulated BMDMs of hTNF.LucBAC. (**a**) Similarly, Nh (black line) and STh (blue line) were treated at 55°C in time course before soluble mouse TNF was measured by ELISA) after LPS-stimulated BMDMs of hTNF.LucBAC. All data are representative of three independent experiments, with triplicate cultures per experiment (N = 3, n = 3) and bars represent standard error of the mean.


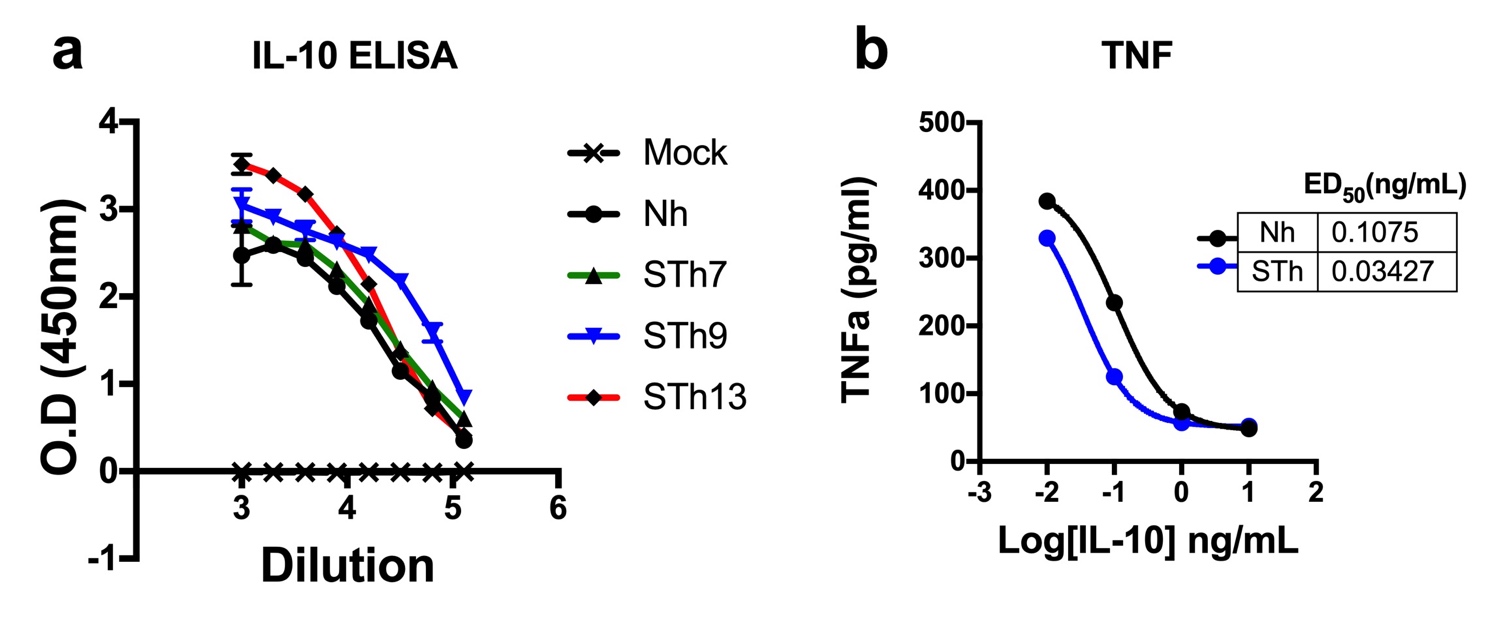


**Figure S2. ELISA detection and functionality of human IL-10 *in vitro*.** (**a)** Nh (black line) and STh7 (green line) STh9 (blue line) and STh13 (red line) were detected ELISA (1/2 dilutions). All data are representative of triplicate wells, and the bars represent standard error of the mean. (**b**) Soluble mouse TNF was measured from the medium 24 hours from BMDMs of the h.TNF.LucBAC reporter mouse after LPS stimulation and co-treating with either Nh (black line) or STm (blue line) in a dose-dependent fashion. All data are representative of two independent experiments, with duplicate cultures per experiment (N = 2, n = 2) and bars represent standard error of the mean.
